# Extrinsically derived TNF is primarily responsible for limiting antiviral CD8+ T cell response magnitude

**DOI:** 10.1371/journal.pone.0184732

**Published:** 2017-09-08

**Authors:** Kylie M. Quinn, Wan-Ting Kan, Katherine A. Watson, Brian J. Liddicoat, Natasha G. Swan, Hayley McQuilten, Alice E. Denton, Jasmine Li, Weisan Chen, Lorena E. Brown, David C. Jackson, Patrick C. Reading, Peter C. Doherty, Katherine Kedzierska, Lukasz Kedzierski, Stephen J. Turner, Nicole L. La Gruta

**Affiliations:** 1 Infection and Immunity Program and Department of Biochemistry and Molecular Biology, Biomedicine Discovery Institute, Monash University, Clayton, Victoria, Australia; 2 Department of Microbiology and Immunology, Peter Doherty Institute for Infection and Immunity, University of Melbourne, Parkville, Victoria, Australia; 3 T cell laboratory, School of Molecular Sciences, La Trobe University, Bundoora, Victoria, Australia; 4 Department of Immunology, St. Jude Children's Research Hospital, Memphis, TN, United States of America; 5 Faculty of Veterinary and Agricultural Sciences, Peter Doherty Institute for Infection and Immunity, University of Melbourne, Parkville, Victoria, Australia; Mayo Clinic Minnesota, UNITED STATES

## Abstract

TNF is a pro-inflammatory cytokine produced by both lymphoid and non-lymphoid cells. As a consequence of the widespread expression of its receptors (TNFR1 and 2), TNF plays a role in many important biological processes. In the context of influenza A virus (IAV) infection, TNF has variably been implicated in mediating immunopathology as well as suppression of the immune response. Although a number of cell types are able to produce TNF, the ability of CD8^+^ T cells to produce TNF following viral infection is a hallmark of their effector function. As such, the regulation and role of CD8^+^ T cell-derived TNF following viral infection is of great interest. Here, we show that the biphasic production of TNF by CD8^+^ T cells following *in vitro* stimulation corresponds to distinct patterns of epigenetic modifications. Further, we show that a global loss of TNF during IAV infection results in an augmentation of the peripheral virus-specific CD8^+^ T cell response. Subsequent adoptive transfer experiments demonstrated that this attenuation of the CD8^+^ T cell response was largely, but not exclusively, conferred by extrinsic TNF, with intrinsically-derived TNF making only modest contributions. In conclusion, TNF exerts an immunoregulatory role on CD8^+^ T cell responses following IAV infection, an effect that is largely mediated by extrinsically-derived TNF.

## Introduction

CD8^+^ T cells are critical for control of viral infections and tumors and their efficient induction requires coordinated signaling through a number of pathways, including T cell receptor (TCR) ligation with peptide in the context of major histocompatibility complex class I (MHC I), costimulatory molecules and cytokines [1]. One of the key effector functions acquired by CD8^+^ T cells upon activation is the ability to produce antiviral and pro-inflammatory cytokines, including IFNγ and TNF. Typically, cytokine production by antiviral CD8^+^ T cells occurs in an hierarchical fashion, with the majority producing IFNγ, and a subset of those producing TNF. Such ‘polyfunctionality’ within a T cell response is used to indicate an increased quality of response, and has been associated with heightened affinity of TCR-pMHCI recognition [2–4].

Tumor necrosis factor (TNF) can substantially influence antiviral CD8^+^ T cell responses. TNF can be expressed as a membrane bound protein (mTNF) or cleaved and released as a soluble protein (sTNF) [5]. Following infection, TNF is expressed by a range of cells, including epithelial cells, natural killer (NK) cells, macrophages, dendritic cells (DCs), CD4^+^ and CD8^+^ T cells [6]. TNF binds to two receptors, ubiquitously expressed TNFR1, and TNFR2, which is more restricted to haematopoetic tissues and is upregulated on activated CD8^+^ T cells [7]. TNFR1 has a death domain to drive apoptosis and it also triggers NFκB driven inflammatory pathways. TNFR2 does not have a death domain and only weakly stimulates NFκB, but coordinated signaling of TNF through TNFR1 and TNFR2 has been shown to have cytotoxic effect on activated CD8^+^ T cells [8, 9], suggesting that TNF:TNFR2 signaling plays an immunoregulatory role. It has been shown that global TNF/TNFR2 signaling inhibits the secondary CD8^+^ T cell response to influenza in the lungs [10]. Studies investigating the role of TNF in anti-influenza immune responses, viral clearance and immunopathology have indicated that TNF is not required for viral clearance in the lungs, but is essential in controlling lung damage [11]. Others reported that sTNF is responsible for limiting the extent of lung injury and this interaction was mediated via TNFR1 [7]. Moreover, the latter study demonstrated that TNF expression is required early during infection to regulate the magnitude of CD8^+^ T cell responses. However, studies with TNF knockout (*Tnf-/-*) mice are limited as this genotype causes defects in the follicular DC network as well as in B cell follicle and germinal centre formation [12]. Consequently, *Tnf-/-* mice have a profound defect in their immune architecture and cellular composition [13]. Therefore, studies using global *Tnf-/-* mice do not allow us to investigate the role of intrinsic TNF produced by CD8^+^ T cells and its role in the infection. Recently, Wortzman *et al*. [14] used an adoptive transfer model of TNF- or TNFR2-deficient transgenic CD8^+^ T cells, and demonstrated that TNF produced intrinsically by CD8^+^ T cells enhanced effector functions and regulated contraction of those cells via TNFR2 signaling.

In our study, we show that the rapid and robust production of TNF after *in vitro* stimulation is dependent on co-stimulation and is associated with changes in histone post-translational modification (PTM) deposition at the *Tnf* gene locus. We also demonstrate that, following intranasal infection with influenza A virus (IAV), global TNF deficiency increased the magnitude of IAV-specific CD8^+^ T cell responses, as measured in the periphery, but did not significantly affect the recruitment of IAV-specific CD8^+^ T cells to the lungs. Moreover, this TNF-mediated attenuation of the IAV-specific CD8^+^ T cell response was found to be largely dependent on extrinsic TNF production, with only a moderate contribution by intrinsic CD8^+^ T cell-derived TNF. These data clearly indicate an immunoregulatory role for TNF during IAV infection, which occurs at the global, rather than local, level and is mediated predominantly by extrinsic TNF production.

## Materials and methods

### Mice

Female 6–12 week old C57BL/6J (WT), ovalbumin transgenic-I (OT-1), *Tnf -/-* and *Tnf -/-/*OT-I mice were bred and housed in specific pathogen-free conditions at the Biomedical Research Facility, Department of Microbiology and Immunology, The University of Melbourne (Parkville, VIC, Aust.). *Tnf -/-* mice [15] were obtained from the Heath Laboratory (University of Melbourne) with permission from the Centenary Institute (Sydney, Australia). Within experiments, mice were age-matched to within 1 week. Mice were killed by CO_2_ asphyxiation using a slow fill rate of 20% per volume per minute. All animal experimentation was conducted following the Australian National Health and Medical Research Council Code of Practice for the Care and Use of Animals for Scientific Purposes guidelines for housing and care of laboratory animals and performed in accordance with Institutional regulations after pertinent review and approval by the University of Melbourne Animal Ethics Committee (S1 File).

### In vitro stimulation and intracellular cytokine staining

Sorted CD8^+^CD44^lo^ T cells stimulated at 2 x 10^4^ cells per well in mAb-coated 24-well plates for up to 72 hours (h) were incubated in the presence of 10 U/mL recombinant human IL-2 (Roche Diagnostics, Mannheim, Germany). The mAb were coated onto Nunc plates overnight in PBS at the following concentrations: anti-CD3ε (clone 145-2C11) at 10 μg/mL; anti-CD8α (clone 53–6.7) at 10 μg/mL; anti-CD11a (clone I21/7.7) at 5 μg/mL; anti-CD28 (clone 37.51) at 10 μg/mL [16]. To permit intracellular cytokine staining, GolgiPlug at a 1:1000 dilution (BD Biosciences, San Diego, CA, USA) was added for the last 5 h of the incubation. After incubation, cells were surface-stained using anti-CD8α-PE (BD Pharmingen; clone 53–6.7), fixed and permeabilised using Cytofix/Cytoperm buffer and 1 × Perm/Wash buffer (BD Biosciences) according to manufacturer’s instructions and stained with anti-IFNγ-FITC (BD Pharmingen; clone XMG1.2) and anti-TNF-APC (BD Pharmingen; clone MP6- XT22) [17]. Cells were then acquired using FACSCalibur flow cytometer (BD Biosciences) and data were analyzed by using FlowJo software (Versions 9&10) (FlowJo LLC, Ashland, Oregon).

### Chromatin immunoprecipitation (ChIP) for histone modifications and RNA polymerase II

Sort purified lymphocytes from LNs and spleen (~5 x 10^6^ cells total) were fixed in 1% formaldehyde, resuspended in ChIP lysis buffer (1% v/v SDS, 10mM EDTA, 50 mM Tris-HCl) and sonicated to generate 200–1000 base pair fragments. Samples were then precleared using Protein A-agarose/salmon sperm DNA (Millipore, 16–157), split into 5 and incubated overnight with either 5 ug anti-H3K27me3, 3 ug anti-H3K4me3 or 4 ug anti-RNA polymerase II (all Invitrogen). A no antibody control and a total input positive control were also included. After washing, all samples (except the ‘total input’) were incubated with Protein A-agarose/salmon sperm DNA with rotation for 1 hour followed by a series of washes in low salt, high salt, lithium chloride, and TE buffers. DNA was eluted before crosslink reversal with 0.2M NaCl at 66°C overnight, followed by protein digestion with proteinase K (Promega). Immunoprecipitated DNA was extracted by phenol: chloroform:isoamyl (25:24:1) extraction and resuspended in HPLC water. For analysis, real-time PCR was used to measure the levels of ChIP-DNA, such that resulting cycle threshold (Ct) values were converted to copy number (#copies = 10^5^/2^Ct-17^) and samples were normalised to their corresponding total inputs with background subtraction (no-antibody control).

### Influenza A virus infection and determination of viral titer

Mice were anesthetized by isofluorane inhalation and infected intranasally with 1x10^4^ PFU of the HKx31 (H3N2) IAV strain in 30 μL of PBS [18]. Weights were monitored daily and mice typically lost <20% of original starting body weight. Viral titres were determined at the indicated timepoints using a plaque assay on monolayers of Madin-Darby Canine Kidney (MDCK) cells. Briefly, lungs were homogenised in 2 mL of incomplete RPMI, the cell suspension was centrifuged and a titration of supernatant was applied to the MDCK monolayers, overlaid, incubated for up to 3 days before plaques were counted.

### T cell adoptive transfer

CD8^+^CD44^lo^ T cells (OT-I or *Tnf -/-/*OT-I) were sort purified (S1 Fig) and 1x10^4^ cells were transferred via intravenous injection into WT or *Tnf -/-* recipient mice. Recipient mice were infected the following day with IAV as described above and spleen and bronchoalveolar lavage (BAL) were taken for tetramer analysis at day 10 after infection.

### Tetramer staining

Spleen or BAL from IAV-infected mice at day 10 were processed to single cell suspensions, red blood cells were lysed and the remaining leukocytes stained. We used PE-labeled MHCI tetramers (D^b^NP_366_ or K^b^PB1_703_) and APC-labeled MHCI tetramers (D^b^PA_224_ or D^b^PB1-F2_62_) (University of Melbourne Tetramer Facility), stained with Fixable Live/Dead AquaBlue viability dye (Life Technologies), blocked with anti- CD16/32 mAb (clone 2.4G2), and stained with anti-CD3ε-PerCPCy5.5 (BD Pharmingen; clone 145-2C11), anti-CD8α-PacBlue (BD Pharmingen; clone 53–6.7) and anti-CD4-AF700 (BD Pharmingen; clone RMA4-5). Cells were acquired on a FACS Canto II flow cytometer (BD Biosciences), and data were analyzed by using FlowJo software (Treestar).

### Statistical analyses

All experiments contained a minimum of 4 mice (4–10) and were repeated at least twice, with similar results. Each symbol represents either data from an individual sample or the mean of multiple samples. Error bars represent standard error of the mean (SEM). Mann-Whitney test with Bonferroni correction for multiple comparisons was used to compare multiple samples and statistically significant differences between groups are indicated as follows: ns = not significant (p>0.05), *p ≤ 0.05, or **p ≤ 0.01.

## Results

### TNF expression kinetics correlate with epigenetic regulation upon TCR stimulation

**Fig 1:**
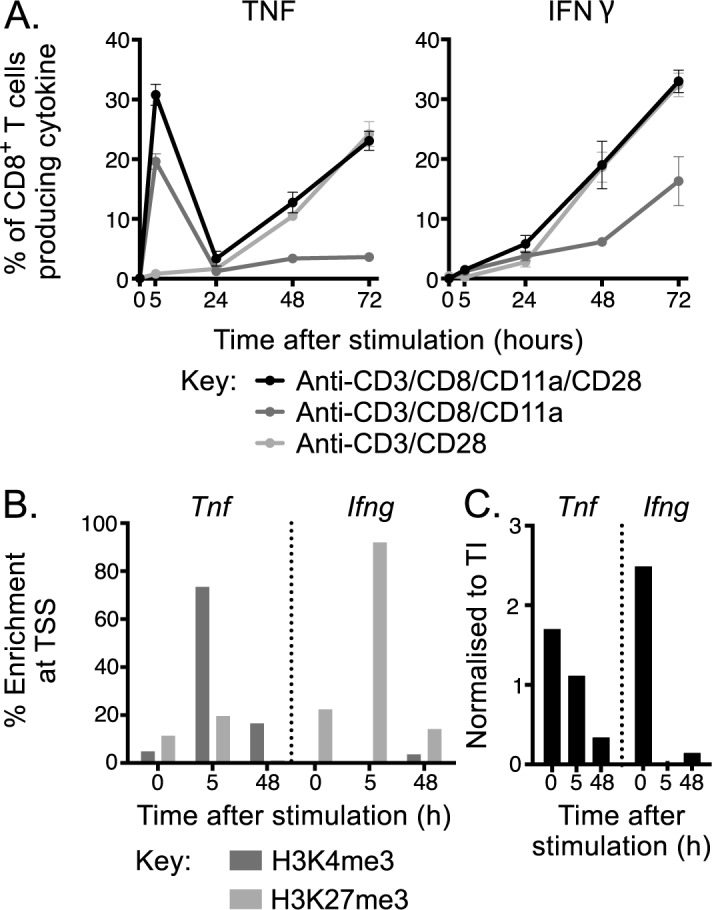
Regulation of TNF expression after TCR stimulation. **A)** Expression of IFNγ and TNF was tracked at 0, 5, 24, 48 and 72 h after TCR engagement with anti-CD3 mAb with co-activation of co-stimulatory signals using intracellular cytokine staining with the frequency of CD8^+^ T cells expressing each cytokine indicated. **B)** Deposition of the permissive mark, H3K4me3, or the repressive mark, H3K27me3, or **C)** RNA polymerase II enrichment at *Ifng* or *Tnf* PPR at 0, 5 or 48 h after stimulation with anti-CD3/CD8/CD11a/CD28. Line graphs represent mean+/-SEM and data are representative of two independent experiments with n = 4.

We first assessed the kinetics of TNF expression following TCR engagement with various levels of co-stimulation, in a population CD8^+^ T cells isolated from pooled lymph nodes of naïve WT mice. Cells were stimulated with anti-CD3 monoclonal antibody (mAb) in combination with co-stimulation from anti-CD28 mAb alone, anti-CD8/CD11a mAbs or anti-CD8/CD11a/CD28 mAbs. At 0, 5, 24, 48 and 72 h after stimulation, cells were harvested and the percentage of CD8^+^ T cells that produced TNF or IFNγ was assessed by flow cytometry (**Fig 1A**). Total cell counts for a distinct but representative experiment were also determined (S2 Fig). There was extremely rapid production of TNF by naïve CD8^+^ T cells after stimulation, with TNF detectable under all stimulation conditions within 5 h. In the presence of anti-CD8/CD11a/CD28 mAb, TNF production appeared biphasic, with an immediate peak at 5 h, followed by a drop at 24 h and then a steady sustained increase that continued out to at least 72 h. The immediate production of TNF appeared dependent on CD8/CD11a co-stimulation, since the only condition that didn’t induce robust immediate TNF production lacked anti-CD11a, while the later sustained production correlated with CD28 co-stimulation, since the condition lacking anti-CD28 costimulation showed relatively poor TNF production at the later timepoints. In contrast, IFNγ^+^ cells, although detectable at 5 h, steadily increased in proportion over the course of the assay under all conditions.

The extremely rapid and robust production of TNF after CD8^+^ T cell stimulation suggested that naïve CD8^+^ T cells are poised for TNF production, as noted previously and supported by the observation that naïve CD8^+^ T cells contain substantial levels of pre-existing TNF mRNA [19]. To determine whether differential kinetics of TNF and IFNγ expression were associated with distinct epigenetic signatures, we stimulated sort purified naïve CD8^+^ T cells as before and performed chromatin immunoprecipitation (ChIP) for histone modifications followed by quantitative PCR targeting the proximal promoter regions (PPRs) of *Ifng* and *Tnf* loci. We targeted histone 3 lysine 4 tri-methyl (H3K4me3) and H3K27me3 as these are associated with permissive or repressive chromatin structure and thereby transcriptional activity or inactivity, respectively [20, 21]. The patterns of histone modification largely correlated with the frequency of cytokine-producing cells, with rapid deposition of the activating H3K4me3 mark at the *Tnf* PPR at 5 h post-stimulation, corresponding to the peak frequency of TNF^+^ CD8^+^ T cells, and a subsequent removal (at 48 h) of the repressive H3K27me3 mark at this site (**Fig 1B**). In contrast, the *Ifng* locus retained enrichment of the repressive H3K27me3 mark at 5 h but showed an increase in the relative enrichment of H3K4me3 at 48 h (**Fig 1B**), corresponding with a substantial increase in the frequency of IFNγ^+^ cells at this time point (**Fig 1A**). Binding of RNA polymerase II (pol II) is a further indication of transcriptional potential. Prior to stimulation the PPRs of *Ifng* and *Tnf* loci appeared poised for transcription, with both showing enrichment of pol II (**Fig 1C**). Upon stimulation pol II binding was only retained at the *Tnf* PPR at 5 h, and was reduced but still present at 48 h. In contrast, pol II was lost from the *Ifng* PPR at 5 h, only becoming detectable again at 48 h, broadly corresponding to the relative kinetics of production of the two cytokines.

Thus, these data suggest that the differential kinetics of TNF and IFNγ production by CD8^+^ T cells after stimulation is, at least in part, controlled by the accessibility of local chromatin structure determined by deposition of key histone modifications at the PPR.

### TNF attenuates virus-specific CD8^+^ T cell responses

**Fig 2:**
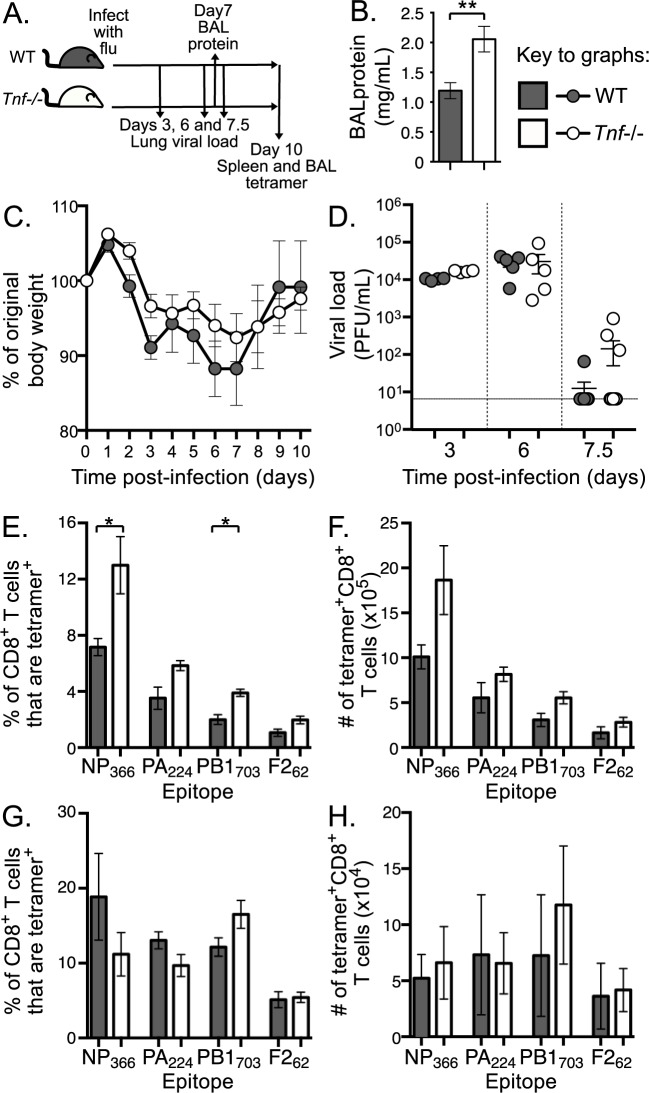
Systemic and local antiviral CD8^+^ T cell responses in the absence of TNF. **A)** Schematic of WT and *Tnf-/-* mouse i.n. infection with 1 x 10^4^ PFU IAV and timeline of subsequent analyses. **B)** Increased local tissue damage as indicated by significantly higher protein concentration in BAL fluid in WT and *Tnf-/-* mice at d 7 after infection. **C)** Percent of original body weight and **D)** viral load in lung tissue after primary influenza infection of WT and *Tnf-/-* mice. Solid line indicates limit of viral detection. **E)** Proportion or **F)** absolute number of CD8^+^ T cells that are IAV-specific using tetramer staining for the indicated epitopes in the spleen at d 10 post primary IAV infection of WT and *Tnf-/-* mice. **G)** Proportion or **H)** absolute number of CD8^+^ T cells that are IAV-specific using tetramer staining in the BAL. Bar graphs and line graphs represent mean+/-SEM and scatter plots represent data for individual mice with bars representing mean+/-SEM. **B/C)** n = 5, **D)** n = 4–10, **E-H)** n = 4–6. * p≤0.05, ** p≤0.01 using Mann-Whitney test with Bonferroni correction for multiple comparisons. Data are representative of **B-D)** 2 or **E-H)** 3–4 independent experiments.

To investigate the role of global TNF on antiviral CD8^+^ T cell responses, we intranasally infected WT and TNF knockout mice (*Tnf*-/-) with the HKx31 (H3N2) strain of IAV (**Fig 2A**). Global loss of TNF in influenza-infected mice led to an increased level of protein in cell-free bronchoalveolar lavage fluid (BAL) on d7 post-infection (**Fig 2B**), typically used as an indication of loss of alveolar barrier integrity and accumulation of protein-rich fluid in the alveolar space [22] but may also reflect increased levels of inflammatory mediators. Despite differences in local lung injury, there was no difference in overall disease severity, as indicated by weight loss (**Fig 2C**), or in viral clearance (**Fig 2D**) between WT and *Tnf*-/- mice during primary influenza infection. We then analysed the endogenous IAV-specific CD8^+^ T cell response in the spleens and BAL of WT and *Tnf*-/- mice at day 10 (d 10) post-infection, which represents the peak of the acute response. To measure CD8^+^ T cell response magnitude, we used MHC class I tetramers, D^b^NP_366_, D^b^PA_224_, K^b^PB1_703_ and D^b^PB1-F2_62_, to identify four influenza-specific populations. This analysis revealed significantly higher proportions (**Fig 2E**) and a consistent trend toward increased absolute numbers (**Fig 2F**) of IAV-specific CD8^+^ T cells in the spleen following infection of *Tnf*-/- mice. However, we found no noticeable difference in the proportions (**Fig 2G**) or absolute numbers (**Fig 2H**) of IAV-specific CD8^+^ T cells in the BAL of infected mice at d 10. Total splenic or BAL-derived CD8^+^ T cell numbers were not different between WT and *Tnf-/-* mice (S3 Fig). This suggests that TNF-mediated attenuation of splenic CD8^+^ T cell responses does not impact the efficiency of IAV-specific CD8^+^ T cell recruitment into the lung.

### Both intrinsic and extrinsic TNF can attenuate IAV-specific CD8^+^ T cell responses

**Fig 3:**
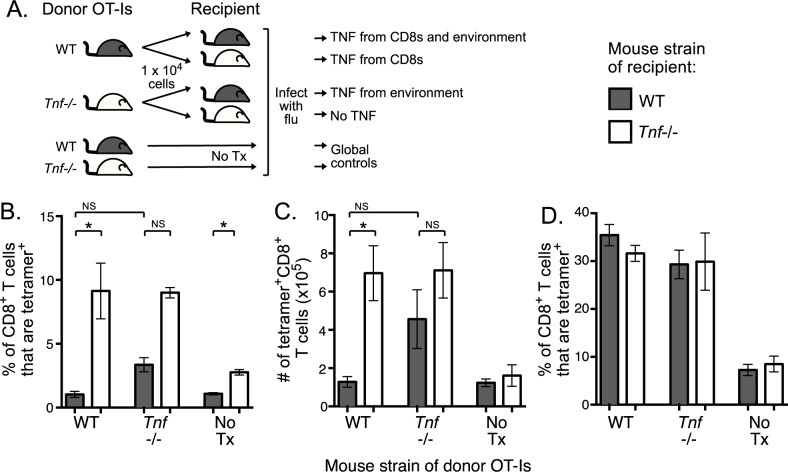
Impact of intrinsic and extrinsic TNF on CD8^+^ T cell responses. OT-I WT or OT-I *Tnf-/-* T cells were transferred into either WT or *Tnf-/-* recipients followed by infection with HKx31-OVA. **A)** Schematic of transfer of 1 x 10^4^ donor OT-I WT or *Tnf-/-* CD8 T cells into WT or *Tnf-/-* mice, alongside WT and *Tnf-/-* control mice that did not receive transferred OT-I cells (No Tx). **B)** Proportion and **C)** absolute number of CD8^+^ T cells that are OVA-specific using tetramer staining in spleen of recipient mice at d 10 after primary IAV infection. **D)** Proportion and CD8^+^ T cells that are OVA-specific using tetramer staining in the BAL. Bar graphs represent mean+/-SEM, data are representative of 3 independent experiments (n = 4–5), * p≤0.05, ** p≤0.01, NS indicates not significant, using Mann-Whitney test with Bonferroni correction for multiple comparisons.

A global absence of TNF affects lymphoid secondary structure and has a profound effect on a range of cell types and their function, especially given that the predominant sources of TNF are macrophages and dendritic cells [13, 23–25]. Thus, to understand the relative impact of intrinsic versus extrinsic TNF on the antiviral CD8^+^ T cell response, we used an adoptive transfer model in which WT or *Tnf*-/- TCR transgenic CD8^+^ T cells expressing a K^b^-restricted ovalbumin (OVA_257-264_)-specific TCR (OT-I) were transferred into either WT or *Tnf*-/- mice, followed by IAV infection (**Fig 3A**) (gating strategy included as S1 Fig). As controls, we included intact WT and *Tnf*-/- mice that did not receive a transfer (No Tx) and we included WT OT-I→WT and *Tnf*-/- OT-I→*Tnf*-/- transfers to control for the effect of cell transfer (**Fig 3A**). Both of these sets of controls were able to recapitulate our observations from Fig 2, with higher proportions (**Fig 3B**) and numbers (**Fig 3C**) of OVA-specific CD8^+^ T cells in the global absence of TNF in the spleen at d10 post-infection. In WT OT-I→*Tnf*-/- transfers, only responding CD8^+^ T cells can produce TNF and the OTI CD8^+^ T cell response was significantly higher than that observed when intrinsic and extrinsic TNF were present in WT OTI→WT transfers (**Fig 3B and 3C**). This suggests that CD8^+^ T cell-derived TNF plays a modest role in attenuating the IAV-specific response. In *Tnf*-/- OT-I→WT transfers, CD8^+^ T cells selectively lack the capacity to produce TNF but the CD8^+^ T cell response magnitude was comparable to that observed in the complete absence of TNF in the *Tnf*-/- OT-I→*Tnf*-/- transfers (**Fig 3B and 3C**). Finally, the loss of global, extrinsic or intrinsic TNF had no impact of the proportion of CD8^+^ T cells that were OVA-specific in the BAL (**Fig 3D**). Again, this finding is consistent with Fig 2 and further reinforces that CD8^+^ T cell- or extrinsically-derived TNF has no impact on local antigen-specific CD8^+^ T cell recruitment. Collectively, these data indicate that extrinsically-derived TNF has the dominant impact on antiviral CD8^+^ T cell responses by attenuating the magnitude in the secondary lymphoid organs, but CD8^+^ T cell-derived TNF can mediate a modest attenuation.

## Discussion

TNF is one of the major mediators of inflammation and its role during influenza infections has been described in several studies [7, 11, 14, 26]. While our work and previous studies in *Tnf-/-* mice demonstrate that TNF is not needed for efficient clearance of virus from the lungs as it is not required for the antiviral activity of CD8^+^ T cells [11, 27], TNF is important for other aspects of antiviral immunity as it displays functional duality, being both pro-inflammatory and immunoregulatory[28]. With regard to its proinflammatory role, TNF has been linked to increased morbidity upon influenza infection, possibly through exacerbation of lung inflammation by promoting recruitment of immune cells [29, 30]. The source of this TNF may be stromal cells, macrophages or dendritic cells but TNF derived from virus-specific CD8^+^ T cells has also been shown to contribute to lung damage [27]. With regard to its immunoregulatory role, TNF may simultaneously limit lung immunopathology by attenuating CD8^+^ T cell responses [31, 32]. In the global absence of TNF, infected mice displayed numerically higher and prolonged IAV-specific CD8^+^ T cell responses in the lungs [11], and this accumulation was specifically linked to the action of sTNF [7]. In addition, it has been shown that TNF produced by activated CD8^+^ T cells can act in an autocrine fashion via TNFR2, to regulate the peak magnitude of the CD8^+^ T cell response and promote contraction [14]. The latter study used the adoptive transfer model of OT-I transgenic cells thus eliminating all other factors that can affect the immune response in global *Tnf -/-* mice.

In this study, we showed, in accordance with previous studies [19], that TNF is produced by CD8^+^ T cells in two waves; namely an immediate burst of TNF that peaks at ~5 h, followed by a more rapid accumulation of TNF production that continues to increase out to 72 h. Early TNF upregulation is consistent with an observation that TNF expression is necessary during priming of T cells in order to attenuate the subsequent effector response [7]. Moreover, early inflammatory events have been shown to control contraction of CD8^+^ T cell responses [33] and TNF has been implicated as a critical factor, sensitizing activated T cells for subsequent apoptosis [34]. Intriguingly, the two waves of TNF production were associated with different patterns of histone modifications, with early TNF production associated with rapid enrichment of the permissive H3K4me3 mark, and later TNF production associated with a loss of the repressive H3K27me3 mark. This is in line with a previous global analysis of epigenetic control of CD8^+^ T cell function [35]. In that study, the gain of H3K4me3 and the loss of H3K27me3 represented two distinct epigenetic mechanisms associated with transcriptional activation, with gain of H3K4me3 correlating with more rapid gene transcription.

Apart from rapid epigenetic changes, we have also previously demonstrated that naïve OTI CD8^+^ T cells contain substantial levels of TNF, but not IFNγ, mRNA transcript [19], which is also likely to contribute to the very early (5h) production of TNF by CD8^+^ T cells after activation. It is difficult to compare these very early epigenetic changes and cytokine production characteristics immediately after *in vitro* stimulation with the events that occur *in vivo*. This is largely because *in vivo* cell division is initiated in very rare, antigen-specific T cell population 2–3 days after infection, which means cells cannot be readily detected *in vivo* until well beyond the timepoints analysed here. However, we have previously shown that OTI cells either activated *in vivo* by infection with HKx31-OVA or *in vitro* by a 5h peptide stimulation, produce TNF protein prior to IFNγ [19], which supports the current findings. Characterization of H3K4me3 and H3K27me3 deposition on the *Tnf* and *Ifng* loci in naïve, effector (10 d) and memory (> 70 d) OTI cells has also been assessed [19]. Although these *in vivo* activated populations are not directly comparable to our analyses immediately after stimulation, naïve OTI cells exhibited elevated H3K27me3 deposition at the *Ifng* compared to *Tnf* locus, as well as detectable levels of H3K4me3 only at the *Tnf*, but not the *Ifng*, locus, consistent with our data and with rapid TNF production after stimulation.

Our analysis of the impact of global loss of TNF on IAV-specific CD8^+^ T cell responses was consistent with previous studies in several respects. Firstly, we found that TNF was not required for efficient clearance of virus from the lung [10, 11], but did reduce lung injury following intranasal infection [7, 11]. Additionally, we found that the absence of TNF resulted in an increased acute IAV-specific CD8^+^ T cell response [7, 14]. Collectively, these data support the idea that TNF plays an immunoregulatory role following IAV infection at a number of points: early after infection (by d 7) to mitigate lung damage, later (d10) to attenuate peak CD8^+^ T cell response magnitude, and thereafter to drive the contraction [14, 36] of the IAV-specific CD8^+^ T cell response.

Our study also contrasted with previous studies that had demonstrated that the loss of TNF increased the severity of influenza infection, as assessed by weight loss, and increased CD8^+^ T cell response magnitude in the lung [7, 11]. We found that an absence of TNF had no impact on disease severity and caused an increase in the proportion and number of IAV-specific CD8^+^ T cells in the spleen, but not in the lung. This suggests that TNF modulates the overall magnitude of the CD8^+^ T cell response but does not affect the recruitment of CD8^+^ T cells to the site of infection. This second discrepancy may be explained by the fact that previous studies investigated the role of TNF in response to relatively virulent strains of IAV, associated with high levels of virus replication and pulmonary inflammation. The HKx31 strain, in comparison, is known to induce a relatively mild infection when delivered intranasally. The requirement for TNF by the CD8^+^ T cell response has previously been shown to differ depending on the inflammatory context, with T cell responses to weak tumor antigens showing greater TNF dependence than a strong anti-LCMV response [23].

Our dissection of the relative impact of CD8^+^ T cell extrinsic versus intrinsic TNF during IAV infection demonstrates that CD8^+^ T cell-derived TNF mediates a modest attenuation of CD8^+^ T cell responses. This is supported by a previous study which concluded that intrinsic TNF potentiated the contraction of the CD8^+^ T cell response after viral clearance [14]. Our study extends these observations by demonstrating that CD8^+^ T cell-derived TNF is not sufficient for full contraction of virus-specific CD8^+^ T cells and that extrinsically-derived TNF was largely responsible for this attenuation in the periphery. Thus, although intrinsically-derived TNF does not appear to play a critical role in this context of mild IAV infection, the fact that its role varies in a context-dependent manner means that an understanding of the kinetics and epigenetic control of TNF expression in CD8^+^ T cells is essential.

In conclusion, TNF is essential for attenuating the peak CD8^+^ T cell response following acute IAV infection, with extrinsically-derived TNF having the greatest impact on response magnitude and quality. This study also found that the effects of TNF were most apparent in the global peripheral response but had little effect on recruitment of CD8^+^ T cells to the site of infection.

## Supporting information

S1 Fig Gating strategy for the identification and isolation of naïve OTI CD8+ T cells. (EPS)

S2 Fig Number of viable cells following in vitro stimulation of CD8+ T cells under various conditions. (EPS)

S3 Fig Total number of CD8+ T cells in spleen and BAL of WT and *Tnf-/-* mice infected 10 d prior with 1 x 10^4^ PFU of HKx31 IAV intranasally. (EPS)

S1 File ARRIVE checklist. (PDF)
